# From Surface Colonies to Internal Contamination: A Comprehensive Investigation of *Alternaria alternata* Growth, Toxinogenesis, and Mycotoxin Migration Dynamics in Cherry Tomato Fruit Matrix

**DOI:** 10.3390/toxins18020070

**Published:** 2026-01-27

**Authors:** Huynh Minh Tan Trinh, Léna Dole, Coline Nazet, Christophe Jourdan, Véronique Martinez, Charlie Poss, Noël Durand, Caroline Strub, Angélique Fontana-Tachon, Sabine Schorr-Galindo

**Affiliations:** 1 Qualisud, University of Montpellier, CIRAD, Institut Agro, IRD, University of Avignon, University of Réunion, UMR QualiSud, F-34398 Montpellier, France; lena.dole@umontpellier.fr (L.D.); coline.nazet@inrae.fr (C.N.); christophe.jourdan@umontpellier.fr (C.J.); noel.durand@cirad.fr (N.D.); caroline.strub@umontpellier.fr (C.S.); angelique.fontana@umontpellier.fr (A.F.-T.); 2 CIRAD, UMR Qualisud, F-34398 Montpellier, France

**Keywords:** cherry tomatoes, *Alternaria alternata*, mycotoxins, toxins migration, food safety

## Abstract

*Alternaria alternata* is a common postharvest mold affecting tomato products, including cherry tomatoes, and causing their contamination with mycotoxins. When consumers encounter moldy fruits, some may remove the visibly contaminated part and consume the rest, to reduce waste. However, the extent to which *A. alternata* toxins migrate beyond visible fungal growth remains unclear, potentially posing health risks. This study investigated (i) the within-fruit migration of *A. alternata* in cherry tomatoes together with the associated mycotoxin production, and (ii) the diffusion of purified *Alternaria* toxins in tomatoes in the absence of any fungal activity. Toxins were quantified using LC-MS/MS, while fungal colonization was assessed through visual inspection and DNA quantification across fruit sections. In the absence of fungal growth, toxins remained largely confined to the spiking site and were degraded over time. In contrast, in inoculated samples, *Alternaria* DNA was detected at notable levels even in sections lacking visible fungal growth, while *Alternaria* toxins were found both in these regions and in lower fruit sections where fungal DNA was below the qPCR detection limit. These findings highlight the limitations of relying solely on visual inspection to assess food safety. A consumer recommendation is proposed to help minimize health risks while reducing food waste.

## 1. Introduction

Tomatoes (*Solanum lycopersicum*) are botanically classified as fruits, yet they are commonly treated as vegetables in both culinary and agricultural contexts. According to FAO statistics, tomatoes ranked among the most widely produced vegetable crops globally in 2023, with an estimated total output of approximately 192 million tons [1]. Their chemical composition is characterized by a high content of nutritionally valuable compounds, including carotenoids, fibers, vitamins, flavonoids, and minerals, all of which contribute to recognized health benefits [2].

However, due to their high water content, delicate epidermal layer, and relatively high sugar and acid levels, tomatoes are particularly vulnerable to microbial spoilage during postharvest storage and distribution [1]. These characteristics, combined with warm and often humid storage conditions prevailing at the retail or household level, render tomatoes a favorable substrate for fungal growth [2].

Numerous fungal contaminants can attack tomato fruits. Examples include *Botrytis cinerea*, *Rhizopus stolonifer*, *Geotrichum candidum*, and *Colletotrichum gloeosporioides*, which cause gray mold, *Rhizopus* rot, sour rot, and anthracnose, respectively, leading to food waste [3]. Species belonging to the genera *Alternaria*, *Fusarium*, and *Penicillium* also contribute to fruit deterioration and food loss; however, they pose an additional concern as they are able to produce mycotoxins [4,5], secondary metabolites that are toxic or potentially harmful to human and animal health. These compounds are colorless, odorless, tasteless [2], and heat-stable, meaning they can persist even after cooking or processing [6]. Critically, mycotoxins are known to diffuse within tomato fruits beyond visibly contaminated areas [7,8], and this diffusion appears to be more pronounced compared to other food matrices such as apples [9]. It is not uncommon for individuals to remove the visibly moldy portion and consume the rest of the fruit, unaware that mycotoxins may have already migrated beyond the visible lesions. In this context, it is necessary to improve our understanding of the risk associated with consuming apparently healthy parts of tomatoes contaminated by mycotoxin-producing fungi. Among the mycotoxin producer fungal genera that affect tomato fruits, *Alternaria* species are the most frequently observed, particularly *Alternaria alternata* being the most frequently isolated [10,11]. In tomatoes, *Alternaria* spp. causes black mold rot, characterized by sunken lesions, softening of tissues, and dark pigmentation due to melanin production [12,13]. It can produce numerous mycotoxins, even in the early stages of contamination [14,15], meaning that fruits showing minimal fungal lesions may already contain high levels of mycotoxins. Its ability to grow and synthesize mycotoxins under refrigerated conditions has been reported, although optimal growth and toxin production generally occur at ambient temperatures, between 20 and 30 °C [16].

The main toxins produced by *Alternaria* spp. are tenuazonic acid (TeA), alternariol (AOH) and alternariol monomethyl ether (AME). TeA is a tetramic acid derivative known for its cytotoxicity due to inhibition of protein release from ribosomes [17]. The cytotoxicity of AOH and AME, which are dibenzopyrone compounds, is associated with their ability to induce a loss of mitochondrial transmembrane potential and trigger cellular apoptosis [17]. AOH and AME also showed genotoxicity as they interfere with topoisomerases, enzymes that play a crucial role in DNA replication and transcription [18].

Although *Alternaria* toxins are not currently regulated, their monitoring is recommended by the European Commission due to their toxicological relevance. Indicative levels have been proposed for processed tomato products: 10 µg/kg for alternariol (AOH), 5 µg/kg for alternariol monomethyl ether (AME), and 500 µg/kg for tenuazonic acid (TeA), but these do not represent consumer safety thresholds [19], as reference toxicological values are lacking. To address this gap, EFSA applied the Threshold of Toxicological Concern (TTC) approach, which estimates risk when hazard data are limited. TTC values were set at 2.5 ng/kg body weight per day for AOH and AME, and 1500 ng/kg body weight per day for TeA [20].

Given the widespread consumption of tomatoes, their susceptibility to fungal contamination, and the toxic potential of *Alternaria* toxins, it is essential to better characterize the relationship between fungal colonization and toxin migration within this fruit matrix. Cherry tomatoes (*Solanum lycopersicum* var. *cerasiforme*), although rarely consumed once visibly spoiled, provide an advantageous experimental model owing to their uniformity and ease of manipulation. In this study, fruits were superficially wounded and inoculated either with *A. alternata* spores or with a commercial toxin solution, enabling a comparison between fungal colonization and toxin diffusion within the fruit tissue. The objective is to quantify toxin migration relative to visible spoilage, thereby generating evidence-based consumer recommendations aimed at reducing food waste without compromising safety.

## 2. Results

### 2.1. Fungal Growth and Mycotoxin Production in Different Media

To establish a baseline toxigenic profile independent of matrix effects, an in vitro assay was first performed to characterize the intrinsic toxin production capacity of the *Alternaria alternata* strain and to justify the presence of TeA, AOH, and AME for subsequent in vivo analyses in the tomato matrix.

**Figure 1:**
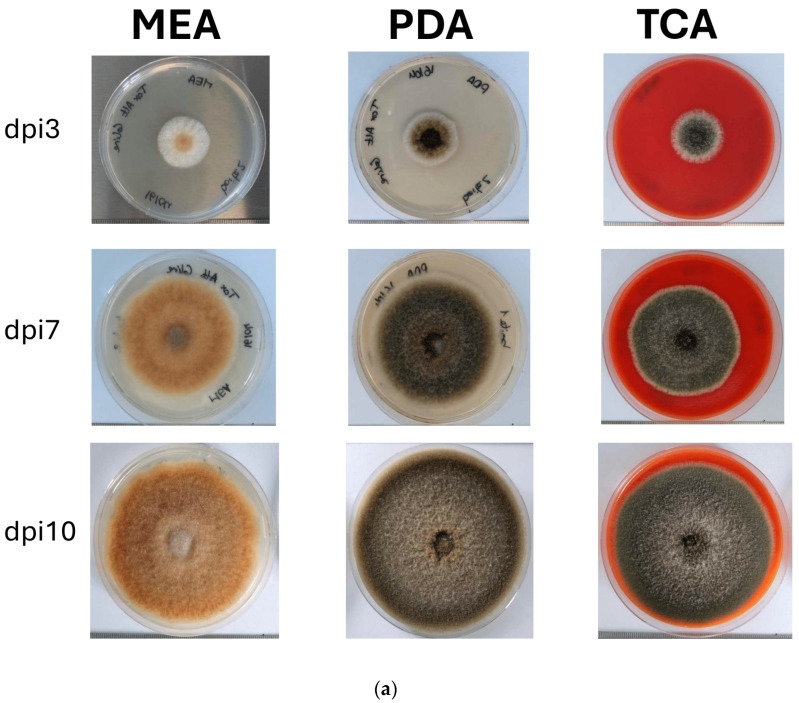

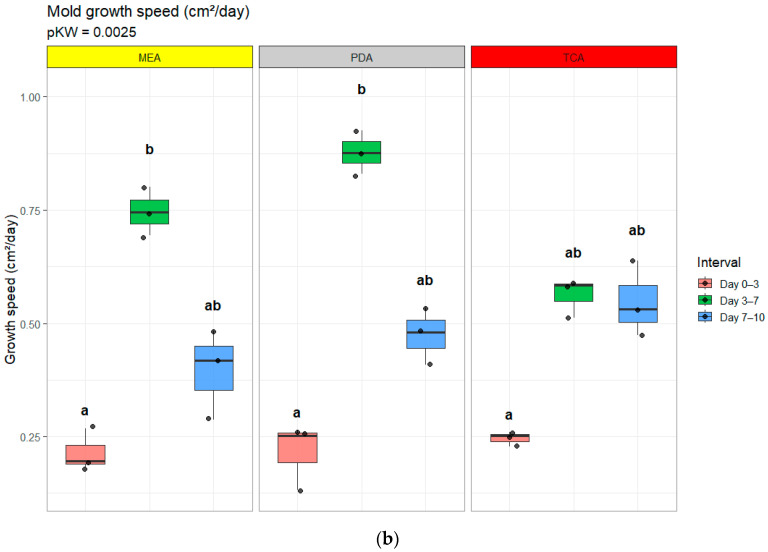
(**a**) Morphological development and (**b**) evolution of growth speed of *Alternaria alternata* on different culture media over a 10-day incubation time at 25 °C. dpi: day post-inoculation; Malt extract agar—MEA; Potato dextrose agar—PDA; Tomato coulis agar—TCA; pKW: *p*-value of the global Kruskal–Wallis test assessing differences in mold growth speed among all medium and time interval combinations; a, ab, b: grouping label indicating statistically significant differences in fungal growth speed (cm^2^/day) among all medium and time-interval combinations, as determined by a Kruskal–Wallis test followed by Dunn’s post hoc test.

On three media PDA (Potato dextrose agar), MEA (Malt extract agar), and TCA (Tomato coulis agar), the *Alternaria alternata* strain, showed distinct morphological and pigmentation profiles depending on the substrate (Figure 1a), with darker colonies on PDA and TCA, while lighter, brownish colonies were seen on MEA.

Fungal growth varied significantly depending on the culture medium (Figure 1b). During the first 3 days, the growth rate was not statistically different among the three media (group a), with an average growth rate of approximately 0.2 cm^2^/day, resulting in colony surface areas of around 0.7 cm^2^. From day 3 to day 10 post-incubation, *A. alternata* exhibited substantial colony expansion on all tested media, with growth kinetics differing according to the substrate. On MEA and PDA, colony surface areas increased from 0.6–0.8 cm^2^ to 3.6–4.0 cm^2^, corresponding to average growth rates ranging from 0.7 to 0.9 cm^2^/day. These conditions were classified in the same statistical group (group b) according to the post hoc test. In contrast, growth on TCA was lower, with colonies reaching approximately 2.8 cm^2^, corresponding to a growth rate of 0.5 cm^2^/day (group ab). Between day 7 and day 10, fungal growth slowed on all three media, notably on PDA and MEA. Growth rates between days 7 and 10 did not differ significantly among the three media and were classified within the same statistical group (group ab), similar to the growth rate observed on TCA between days 3 and 7 (ranging from 0.5 to 0.6 cm^2^/day). By day 10, colonies on all three media had nearly reached full plate coverage.

The toxigenic profile of *A. alternata* varied across culture media (Table 1). Among the three toxins analyzed, TeA was consistently predominant in terms of concentration. From day 3 to day 10, TeA production peaked on PDA, reaching 30.1–68.9 µg/cm^2^, and was not significantly different from that observed on MEA (31.2–49.2 µg/cm^2^). In contrast, TCA supported a lower but relatively stable TeA production throughout the incubation period, with concentrations ranging from 6.9 to 35.6 µg/cm^2^ between day 3 and day 10. In contrast to TeA, AOH and AME exhibited delayed production. From day 3 to day 7, AOH concentrations remained low and stable across all three media (0.01–0.38 µg/cm^2^). By day 10, AOH production increased significantly on MEA, reaching concentrations of 19.8–26.8 µg/cm^2^. In contrast, AOH production on PDA remained consistently low throughout the incubation period, with concentrations ranging from 0.09 to 0.89 µg/cm^2^ from day 3 to day 10 and no significant temporal variation. On TCA, increase in AOH production was measured from day 3 to day 10, ranging from 0.01 to 1.1 µg/cm^2^, but still inferior to the production on MEA. AME production remained low over the entire incubation period, with a significant increase observed only at day 10 on MEA (9.94–15.56 µg/cm^2^) compared with non-detected levels at day 3. On PDA and TCA, AME concentrations remained low throughout incubation, increasing only slightly over time from non-detected levels to a maximum of 0.46 µg/cm^2^.

### 2.2. Inoculation Site Determines the Directionality and Depth of Fungal Migration in Tomato Tissues

**Figure 2:**
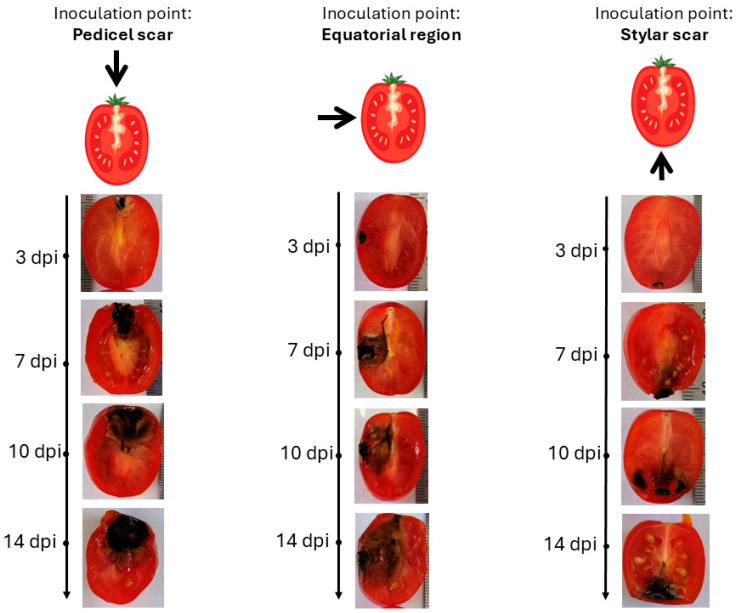
Migration of *Alternaria alternata* in tomatoes over time in 25 °C based on the inoculation point. (dpi: Post-inoculation day). The arrow indicates the inoculation point.

To determine the optimal inoculation site for tracking *Aternaria alternata* migration, cherry tomatoes were inoculated at the pedicel scar, equator, and stylar scar, as the fungal progression can be varied by inoculation point (Figure 2).

Inoculation at the pedicel scar and equatorial region resulted in the most rapid and extensive colonization. From the pedicel scar, the fungus progressed along the vascular bundles, reaching the columella, placenta, and associated seeds by day 10, with near-complete colonization of the internal locular tissues observed by day 14. At the equatorial region, fungal growth initially spread laterally within the pericarp before redirecting toward the pedicel through the vascular tissues, ultimately invading the columella and placental regions, following a similar internal colonization pattern to that observed from the pedicel site by day 14. In contrast, inoculation at the stylar scar exhibited delayed progression, with minimal internal development prior to day 10 and only limited invasion of the central locular tissues by day 14.

Due to its early, direct vascular colonization, the pedicel scar was chosen for further studies on fungal migration and toxin diffusion.

### 2.3. Visual and Molecular Assessments of Fungal Growth in Cherry Tomato Tissue

*Alternaria alternata* inoculated at the pedicel of cherry tomatoes and incubated at 25 °C became macroscopically visible by day 3 post-inoculation, covering a projected surface area of 0.047 cm^2^, corresponding to a volumetric estimate of 0.027 cm^3^ of internal propagation, representing about 0.86% of the total fruit volume. Between days 7 and 10, the colony expanded markedly, increasing from 0.848 cm^2^ (0.226 cm^3^; 15.55% fruit volume) to 1.536 cm^2^ (0.598 cm^3^; 28.15% fruit volume) of the fruit, reflecting an 81.1% increase in visible fungal colonization. By day 14, the external colony covered a surface of about 1.7 cm^2^ around the pedicel, and internal mycelial growth had progressed to 0.95 cm^3^, representing 44.71% of the total fruit volume. Extensive penetration into the fruit parenchyma was observed, accompanied by pronounced softening and tissue disintegration, particularly around the inoculation site, indicating advanced fungal invasion (Table 2). In contrast, at 8 °C, *A. alternata* growth was strongly inhibited. After 14 days, the visible colony area remained limited to 0.036 cm^2^, with an estimated internal volume of 0.027 cm^3^ (0.65% of the fruit volume), more than 97.9% lower than that observed at 25 °C (Table 2). These results demonstrate that cold storage markedly delays *A. alternata* development and significantly limits both surface and internal colonization of cherry tomatoes.

Following the visual assessment of the in vivo kinetic model, a system of morphological contamination levels was established (Table 3):

1. **Level 1—Early contamination stage:** Limited visible mold was observed on the fruit surface, typically around the pedicel, although minor internal fungal growth could also be detected.
2. **Level 2—Spreading stage:** Signs of surface darkening became clearly apparent, while internal colonization extended up to 1 cm from the inoculation site, representing approximately 16% of the fruit’s total volume.
3. **Level 3—Invasive stage:** Clear external mold symptoms were visible, and internal growth extended up to 1.5 cm deep, corresponding to approximately 28% of the total fruit volume. About half of the fruit tissue became softened and wrinkled around the visibly colonized surface.
4. **Level 4—Rotten stage:** The fruit appeared visibly softened, blackened and wrinkled, with extensive internal fungal invasion reaching 2 cm deep, affecting nearly 60% of the fruit’s internal volume.

### 2.4. In Vivo Molecular Assessment and Production of Mycotoxins by Alternaria alternata in Cherry Tomatoes

**Figure 3:**
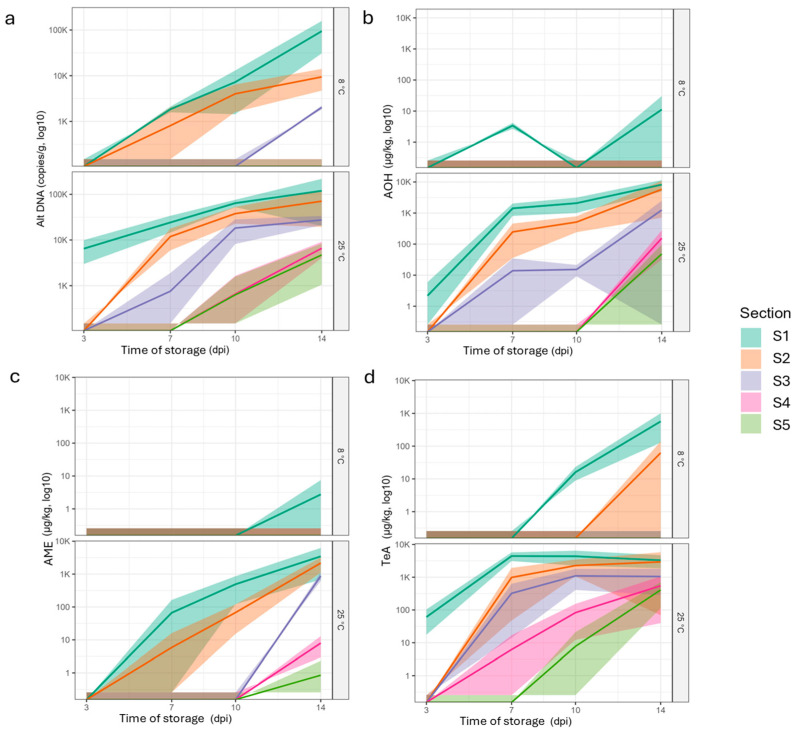
Evolution of the concentration of (**a**) *Alternaria* DNA, (**b**) AOH, (**c**) AME, and (**d**) TeA across tomato sections ranging from S1 (inoculation point) to S5 (0.5–1 cm per section) over the 14 days of storage. In the graphs, lines represent connected means, and the surrounding bands indicate standard deviation (*n* = 3 × 6 fruits). The data are represented in a log10 scale.

Quantitative PCR (qPCR) analysis was performed across tissue layers within and beyond the visibly colonized areas to quantify fungal DNA present in the cherry tomato matrix (LOQ = 10^3^ Alt DNA copies/g matrix) (Figure 3a). Two inoculation models were investigated under different storage temperatures: 25 °C (ambient storage) and 8 °C (cold storage), to reflect conditions relevant to household consumer practices.

At 25 °C, day 3 post inoculation, *A. alternata* DNA was measured in the first section of the inoculated fruit, with a mean concentration of 6464 Alt DNA copies/g matrix, corresponding to a small visible colony limited to 0–0.5 cm from the inoculation point, representing an early contamination profile. From day 7 onward, fungal proliferation increased, as fungal DNA was measured in section 3 (mean 2270 Alt DNA copies/g matrix), approximately 1.5 cm from the inoculation site and still within the visible colony. By day 10, *A. alternata* DNA was detected in sections 4 and 5 (mean 1900 Alt DNA copies/g matrix), extending beyond the visibly colonized area, which remained limited to sections 3 and corresponding to distances of up to 2.5 cm from the inoculation point. By day 14, the same spatial trend was observed, but with higher DNA levels, ranging from 4226 to 9853 Alt DNA copies/g matrix. At 8 °C, Alt DNA was detected at quantifiable levels later than at 25 °C, first appearing in sections 1 and 2 at day 7, with a mean concentration of 1845 Alt DNA copies/g matrix across these sections. Although the visible colony remained restricted to section 1 (0–0.5 cm from the inoculation point), fungal DNA was detected in section 2 (0.5–1 cm) at day 10 (mean 2464 Alternaria DNA copies/g matrix) and in section 3 (1–1.5 cm) at day 14 (mean 2026 Alt DNA copies/g matrix).

Toxin production by *Alternaria alternata* (TeA, AOH, AME) in 25 °C and 8 °C incubation was also evaluated across the different sections of the cherry tomato matrix (Figure 3b–d).

At 25 °C, AOH production was low at day 3 and was quantified only in the first section, with a mean concentration of 7 µg/kg matrix (Figure 3b). By day 7, AOH was measured up to section 3 of the cherry tomato, within the visible colony, with a mean concentration of 21 µg/kg matrix. At day 10, AOH was detected within the visibly colonized area (sections S1–S3), with concentrations ranging from 11 to 3074 µg/kg matrix, but remained undetected outside the visible colony. By day 14, AOH was detected beyond the visibly colonized zone, which remained limited to section 3, with concentrations ranging from 8 to 102 µg/kg matrix in sections 4 and 5. The detection of Alt DNA was consistently associated with the presence of toxins, even at low DNA levels and in the absence of visible fungal growth. At 8 °C, AOH production was limited, with a mean concentration of 3 µg/kg matrix detected at day 7 and confined to the inoculation layer (section 1) of the cherry tomato. AOH was not detected thereafter, except for a weak signal observed in section 1 at day 14 (34 µg/kg matrix), which was detected in only one replicate.

AME production at 25 °C was initially limited compared with AOH (Figure 3c). AME was first detected at day 7 within the visibly colonized area (sections 1 and 2), with concentrations ranging from 8 to 179 µg/kg matrix. By day 10, AME concentrations increased but remained confined to sections 1 and 2, within the visible colony zone, which extended up to section 3, with concentrations ranging from 10 to 866 µg/kg matrix. By day 14, AME production increased markedly, with concentrations ranging from 690 to 8856 µg/kg matrix within the visibly colonized area limited to section 3. Day 10 also marked the first detection of AME beyond the visible colony zone, with concentrations ranging from 3 to 14 µg/kg matrix in sections 4 and 5. At 8 °C, AME concentrations were nearly undetectable, with the exception of a single detection at day 14 in section 1 (8 µg/kg matrix), observed in only one replicate.

The TeA production profile was markedly more intense compared with those of AOH and AME (Figure 3d). At 25 °C, 3 days post-inoculation, a mean TeA concentration of 61 µg/kg matrix was detected at the inoculation site (section 1). By day 7, when the visible colony was limited to section 3 TeA was already detected within the visibly colonized area, with a mean concentration of 322 µg/kg. At day 10, high TeA concentrations were still detected within the visibly colonized zone (sections 1–3), ranging from 409. to 5920 µg/kg matrix. On the same day, TeA was also detected outside the visible colony (sections 4 and 5), with concentrations ranging from 23 to 164 µg/kg matrix. By day 14, TeA showed extensive migration throughout all tomato sections, with concentrations in section 5 (0.5–1 cm beyond the visible colony boundary) ranging from 72 to 620 µg/kg matrix. At 8 °C, TeA was first detected at day 10 post-inoculation, with a mean concentration below LOQ, confined to section 1 at the inoculation site, where visible fungal growth remained limited. At day 14, concentrations ranging from 38 to 1058 µg/kg matrix in sections 1 and 2, extending beyond 1 cm from the visibly colonized area, in the inoculation site, which remained restricted to section 1.

### 2.5. Spatial Toxin Production Activity Based on Toxin to DNA Ratios

**Figure 4:**
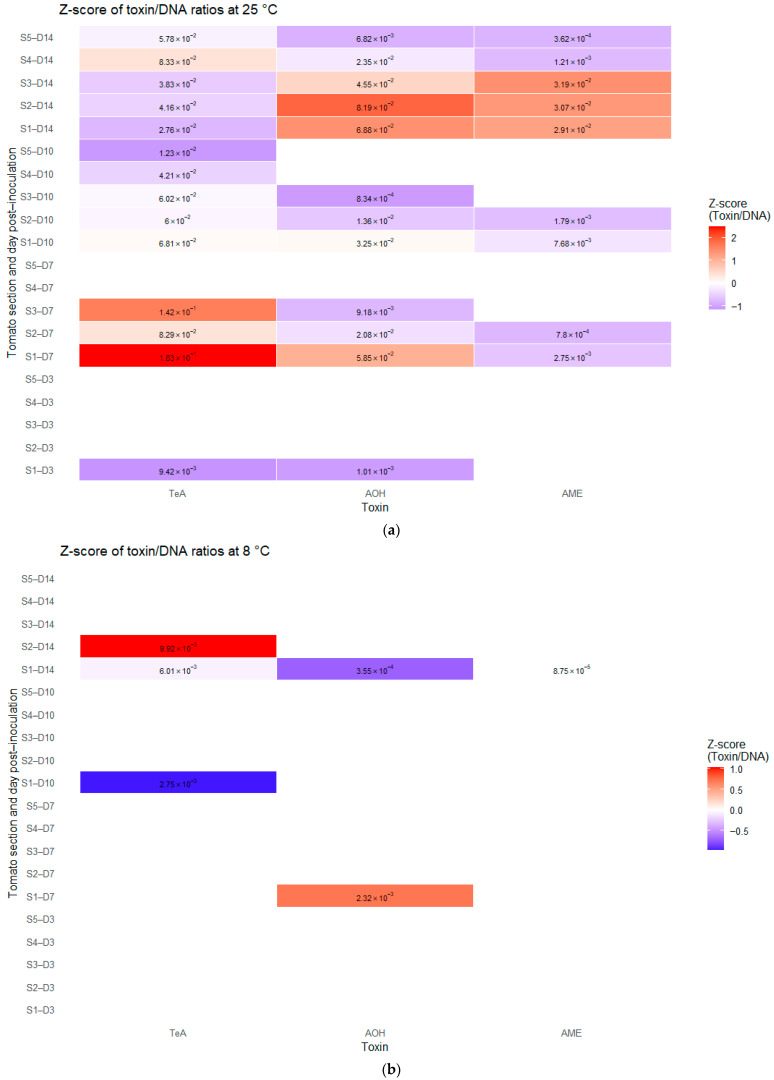
Relative toxigenic activity of *Alternaria alternata* (TeA, AOH, AME) across cherry tomato sections, expressed as z-scores of toxin/DNA ratios at 25 °C (**a**) and 8 °C (**b**). Blank cells correspond to values below the limit of quantification (LOQ) for either fungal DNA or the corresponding toxin.

The z-score of the toxin to DNA ratio was used to assess toxin production activity normalized to fungal biomass across the different cherry tomato sections (Figure 4).

At day 3, incubated at 25 °C (Figure 4a), toxin production was proportional to fungal biomass, with very low toxin-to-DNA ratios for TeA and AOH (9.42 × 10^−3^ and 1.01 × 10^−3^, respectively). By day 7, section 1 displayed markedly elevated z-scores for both TeA (1.83 × 10^−1^) and AOH (5.85 × 10^−2^), indicating an intensification of fungal secondary metabolism. Notably, for TeA only, sections 2 and 3 also showed high toxin-to-DNA ratios (8.29 × 10^−2^ and 1.42 × 10^−1^, respectively), compared with the corresponding sections for AOH and AME. On the same day (day 7), limited AME production activity was also detected in sections S1 and S2, with negative z-scores and low ratio of 2.75 × 10^−3^ and 7.80 × 10^−4^, respectively. At day 10, z-scores in section 1 for both TeA and AOH decreased but remained positive (6.81 × 10^−2^ and 3.24 × 10^−2^, respectively), suggesting a reduced but persistent toxin production activity. In contrast, all downstream layers (S2 to S5) exhibited negative z-scores, ranging from −6.00 × 10^−2^ to −1.23 × 10^−2^, indicating that active toxigenic production has not yet occurred or weak in this part of the fruit. The toxin to DNA ratio patterns for AME remained similar to those observed at day 7, with a decrease in the AOH z-score in section S1 (from 5.85 × 10^−2^ to 3.25 × 10^−2^). By day 14, the first three sections from the inoculation point (S1, S2, and S3) exhibited high z-scores associated with elevated fungal activity in AOH and AME production, ranging from 4.55 × 10^−2^ to 8.19 × 10^−2^. However, this activity was no longer observed beyond section 3, as sections 4 and 5 displayed low to negative z-scores (−6.82 × 10^−3^ to −2.35 × 10^−2^), indicating the presence of toxins in zones with low fungal DNA levels. TeA production at day 14 appeared more stable overall, as sections 1 to 5 (with the exception of S4) showed low z-scores ranging from 2.76 × 10^−2^ to 5.78 × 10^−2^. Notably, section S4 exhibited a higher z-score (8.33 × 10^−2^), consistent with TeA production activity associated with a newly colonized fungal zone, as fungal DNA was detected in this section.

At 8 °C (Figure 4b), the z-score profiles indicated overall lower fungal activity compared with those observed at 25 °C. At day 3, z-scores could not be determined for any layer or toxin, reflecting the absence of detectable toxin production relative to fungal biomass. By day 7, only AOH activity was detected, although limited to section 1, a positive z-score (2.32 × 10^−3^) was observed, corresponding to the onset of toxin production. At day 10, AOH production was no longer detected, with a negative z-score for the TeA/DNA ratio (2.75 × 10^−3^) appeared in S1, indicating minimal TeA production activity. AME production remained negative at day 10 at 8 °C. By day 14, increased toxigenic activity was observed, although it remained markedly lower than that observed at the same time point at 25 °C. For TeA, a negative z-score was detected in section 1 (6.01 × 10^−3^), while a higher z-score was observed in section S2 (9.92 × 10^−3^), suggesting deeper fungal activity within the matrix. In contrast, AOH and AME activities remained weak, with near-zero to negative z-scores and were restricted to section S1 (3.55 × 10^−4^ and 8.75 × 10^−5^, respectively).

### 2.6. Diffusion of Toxins in Tomato Sections After Artificial Toxin Contamination

To specifically assess the intrinsic diffusion behavior of *Alternaria* toxins in the absence of fungal growth, a spiking experiment was performed in which the mycotoxins TeA, AOH, and AME were injected into cherry tomatoes, and their spatial distribution was monitored over time (3, 7, 10 and 14 days) during incubation at 25 °C and 8 °C.

**Figure 5:**
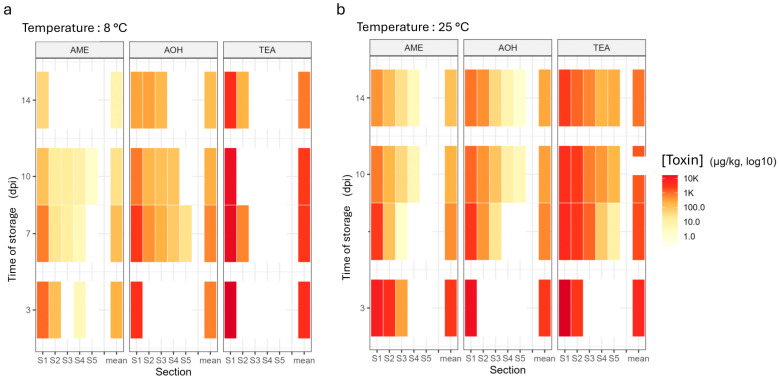
Heatmap depicting the evolution of *Alternaria* toxin concentrations (AOH, AME, and TeA) across tomato sections, from the spiking point (S1) to (S5), over a 14 days storage period at (**a**) 8 °C or (**b**) 25 °C. Color intensity represents the mean concentration for *n* = 3 × 6 fruits. The detection of toxins in sections below S1 indicates diffusion, while the progressive decrease in color intensity across days post-inoculation reflects diffusion or degradation. The final column represents an estimate of the mean toxin concentration at the whole-fruit scale.

At 25 °C (Figure 5b), from day 3 post-spiking, TeA concentrations were detected mainly in sections 1 and 2 near the spiking point, ranging from 8426 to 51,187 µg/kg matrix. By day 7, diffusion toward distal sections was observed, with a mean TeA concentration of 23 µg/kg matrix detected in section 5, approximately 2 cm from the spiking point. One notable observation is that the mean TeA concentration in the whole cherry tomato at day 7 was lower than at day 3 (mean values of 9583 and 31,132 µg/kg, respectively), corresponding to an approximate 69% decrease in total concentration. The same trend persisted at day 10, with higher TeA concentrations detected in section 5 (534 µg/kg matrix). A 20% decrease in the mean toxin concentration in the whole fruit was observed at day 10 compared with day 7 (from a mean of 9583 to 7700 µg/kg). By day 14, this pattern remained evident, although TeA concentrations in section 5 decreased to a mean of 236 µg/kg matrix, accompanied by a progressive decrease in concentrations from proximal sections (S4 to S1) toward the spiking point. At this stage, compared with day 10, a 42% decrease in the mean toxin concentration of the whole matrix was also observed, declining from 7700 to 4492 µg/kg.

At 8 °C (Figure 5a), TeA diffusion was markedly reduced. Across all sampling times, TeA was detected only in the first two sections adjacent to the spiking point. At day 3 post-spiking, a mean concentration of 16,909 µg/kg matrix was detected exclusively in section 1. By day 7, TeA was detected in section 2 (mean 566 µg/kg matrix) and remained high in section 1 (mean 10,769 µg/kg matrix). The total mean TeA concentration in the fruit scale at day 7 (11,335 µg/kg) decreased by 33% compared with day 3 (16,909 µg/kg). At day 10, TeA concentrations in section 1 decreased to a mean of 9455 µg/kg matrix, with no detectable TeA in section 2. A further decrease in the total mean TeA concentration of the whole matrix was observed from day 7 to day 10, corresponding to a 16% loss (from 11,335 to 9555 µg/kg). By day 14, TeA was again detected in section 2 (mean 219 µg/kg matrix), concomitant with a further decrease in section 1 (mean 3018 µg/kg matrix). Compared with day 10 (9555 µg/kg), the mean TeA concentration in the whole fruit at day 14 (9455 µg/kg) showed only a minimal decrease.

At 25 °C (Figure 5b), AOH followed a diffusion pattern similar to that of TeA. At day 3 post-spiking, AOH was detected only in section 1, with a mean concentration of 13,756 µg/kg matrix. By day 7, diffusion was limited to section 3, approximately 1.5 cm from the spiking point, with a mean concentration of 30 µg/kg matrix. The total mean AOH concentration across all matrix sections at day 7 was reduced by 79% compared with day 3, decreasing from 13,756 to 2903 µg/kg. At day 10, AOH was detected at low concentrations in sections 4 and 5, ranging from 11 to 32 µg/kg matrix. A 43% reduction in the mean total AOH concentration of the whole matrix was observed from day 7 to day 10 (from 2903 to 1653 µg/kg). By day 14, AOH remained detectable in sections 4 and 5, with concentrations ranging from 4 to 8 µg/kg matrix. Although AOH concentrations were lower than those of TeA, they followed the same overall trend of decreasing toxin concentration with increasing distance from the spiking point. At day 14, the total mean AOH concentration across all sections showed a further slight decrease compared with day 10, declining from 1653 to 1373 µg/kg.

At 8 °C (Figure 5a), AOH diffusion appeared more persistent than TeA diffusion. At day 3, AOH was still confined to section 1 (mean 3053 µg/kg matrix). From day 7 to day 10, AOH was detected up to section 5, with concentrations ranging from 93 to 149 µg/kg matrix. A further loss in the mean AOH concentration of the whole matrix was observed from day 3 to day 7 and continued to day 10, decreasing from 3054 to 2699 and then to 1146 µg/kg, respectively. By day 14, AOH detection was again limited to section 3 from the spiking point, accompanied by a decrease in concentrations in section 1, which ranged from 223.2 to 409 µg/kg matrix. The mean total AOH concentration in the fruit further decreased from 1146 µg/kg at day 10 to 781 µg/kg at day 14.

AME, at 25 °C (Figure 5b), exhibited earlier diffusion compared with both TeA and AOH, as AME was already detected in section 3 at day 3 post-spiking, with a concentration of 900 µg/kg matrix. By day 7, AME detection remained limited to section 3, but concentrations decreased markedly, with only 4 µg/kg matrix detected. Following the same trend as TeA and AOH, the mean total AME concentration in the whole matrix decreased from day 3 to day 7, declining from 13,012 to 2451 µg/kg. At day 10, AME was detected at low concentrations in section S4 (14 µg/kg matrix). The mean total AME concentration across all sections decreased by 61% from day 7 to day 10, declining from 451 to 962 µg/kg. By day 14, although still be detected in section S4, AME was observed across all proximal sections (1 to 3, and 5), with mean concentrations ranging from 4 to 600 µg/kg matrix. This was accompanied by an overall decrease in AME concentrations compared with those measured in the same sections at day 10, with mean values declining from 4501 to 390 µg/kg of matrix.

At 8 °C (Figure 5a), AME was detected in section S2 as early as day 3, with concentrations ranging from 9 to 4000 µg/kg matrix. Low AME concentrations were also detected on the same day in section S4 (mean 19 µg/kg matrix), despite the absence of detectable AME in the intervening section S3. By day 7, AME was detected in section S4 (18 µg/kg matrix), while section S3 showed detectable AME at a mean concentration of 16 µg/kg matrix. The mean total AME concentration in the whole fruit decreased slightly from 884 to 705 µg/kg between day 3 and day 7. At day 10, AME was detected down to 2.5 cm from the spiking point (section S5), with a mean concentration of 9 µg/kg matrix. Following the same trend, the total mean AME concentration in cherry tomatoes decreased by a further 72% from day 7 to day 10, declining from 705 to 198 µg/kg. By day 14, AME was detected only in section 1 at the spiking site (mean concentration of 60 µg/kg of matrix), with no detectable AME in downstream adjacent tissues, corresponding to an overall decrease of 69% in total mean concentration from day 10.

## 3. Discussion

This study highlighted the influence of the growth substrate on the toxinogenesis of *Alternaria alternata*. TeA production was predominant in vitro across the three media tested, with modified MEA, PDA, and TCA (which is a synthetic tomato-based medium). This predominance has been consistently reported in the literature, both in in vitro and in vivo culture studies [21,22,23], as well as in analyses of contaminants in food products [17]. No significant differences were observed in the amounts of TeA produced among the different media. Similarly, bibliographic data indicate that TeA production appears to be only weakly affected by the chemical composition of the substrate and remains high at the initial pH values of the three media tested, which ranged from 4 to 5.5 [24]. The only notable variation was observed for the tomato-based medium (TCA), in which TeA production (day 7) began earlier than on MEA and PDA. This early activation was also observed during growth on fruit (Figure 4a). Such an early onset of TeA production on a tomato-based substrate has not been previously reported in the literature. To the other studied *Alternaria* toxins, the main difference was observed on the modified MEA medium, where a particularly high late production (day 10) of AOH and AME was detected. This phenomenon was not observed on PDA or TCA. According to the literature, high levels of AOH and AME production have been associated with various aspects of the chemical composition of the substrate. First, AOH and AME production appears to be conditioned by the carbon-to-nitrogen ratio of the substrate, but according to specific thresholds, reflecting fine regulatory control rather than a linear response [24,25]. Second, the initial pH of the medium influences toxin production, with an optimum at pH 4.5 [26]. However, the initial pH values of the PDA and TCA media are 5.5 and 4, respectively, further from the reported optimum than that of the modified MEA medium (pH 4.7). In addition, the nature of nitrogen and carbon sources also influences the synthesis of AOH and AME: phenylalanine, ammonium nitrate salts, or aspartate have been shown to strongly promote their production [24]. However, these compounds are not expected to be present at higher levels in the modified MEA medium compared to the other media tested [27,28,29]. Regarding carbon sources, the modified MEA medium is composed of 20 g/L glucose and 30 g/L malt extract rich in maltose; the PDA contains 20 g/L glucose and 4 g/L potato powder rich in starch; and the TCA contains 32 g/L carbohydrates, mainly fructose and glucose. However, the literature indicates that glucose, rhamnose, and sodium acetate more strongly stimulate AOH production [26], whereas only glucose is present in substantial amounts across all three media. This late production of AOH and AME, although absent on TCA, was observed in tomato fruit, with a marked increase in the toxin-to-fungal DNA ratio at day 14 (Figure 4a). This finding suggests that the risk of contamination by these two toxins increases substantially once the infection is well established. One study reports that AOH and AME synthesis is initiated when nitrogen salts in the medium are depleted under in vitro conditions [26]. However, this explanation alone is insufficient to account for the differences observed between MEA and the PDA/TCA media, since PDA is, a priori, the medium expected to contain the lowest nitrogen content.

In vivo experiments revealed that *Alternaria alternata* colonization can result in detectable mycotoxins well beyond the visibly colonized areas of cherry tomatoes. Notably, at 25 °C, TeA, AOH and AME were detected up to 2.5 cm from the inoculation site, in the rotten level, while the visible fungal colony extended only about 1.5 cm from the inoculation point. This suggests that internal fungal progression, possibly below the visual detection threshold or facilitated by tissue degradation, enables toxin accumulation deeper within the fruit. These findings indicate that even when visible mold is limited to a small area, substantial internal contamination may occur, potentially rendering most of the fruit unfit for consumption. At 8 °C, both fungal development and mycotoxin production were delayed and reduced. Nevertheless, TeA was detected in tissues located 1 cm from the inoculation site, beyond the visible colony (0.5 cm from inoculation point) and exceeded regulatory limits, while still in the early contamination morphological stage, indicating that cold storage may slow but does not eliminate toxin risk. This is consistent with previous findings that certain fungi can continue to metabolize and produce secondary metabolites under cold stress [30], but with TeA instead of AOH. Further work should explore longer storage periods under low temperatures to determine whether subthreshold fungal presence can result in cumulative toxin migration over time. Compared to many previous studies, the *Alternaria* toxin profile observed in this work follows a different pattern. In studies such as those by Qin et al. (2022) [14], Jürg Noser et al. (2011) [31], María Luisa Maldonado Haro et al. (2023) [32], and Terenzio Bertuzzi et al. (2021) [33], TeA is consistently highlighted as the predominant mycotoxin, with concentrations significantly higher than those of AOH and AME. Similarly, in our study, TeA production at 25 °C from day 3 to day 10 was dominant. However, by day 14, AOH concentrations surpassed those of TeA, with a mean value of 15,434 µg/kg detected in whole fruit, compared to 8228 µg/kg for TeA. In Qin et al.’s study [14], TeA levels peaked at day 11 at 25 °C and subsequently declined. In contrast, our in vivo model did not show such a decrease in TeA from day 3 to day 14. Moreover, the increase in AOH concentration at day 14 suggests enhanced metabolic activity, a trend also observed for AME. Although AME levels remained lower than TeA at day 14 (mean: 6515 µg/kg), the z-score analysis indicates a converging trend among the three toxins (Figure 4a). Overall, toxin concentrations in our study were more closely aligned across the three compounds, unlike the stark differences reported in previous works [14,31,32,33], where TeA concentrations were often an order of magnitude higher than AOH and AME. This shift may be attributed to differences in the *Alternaria* strain used, the specific tomato cultivar, or matrix composition. Future studies should investigate fungal metabolic profiles to better understand these variations.

Importantly, when tomatoes were spiked with purified toxins, passive migration was observed. At both 25 °C and 8 °C, TEA, AOH, and AME migrated from the injection site and progressively degraded over time, possibly by interacting with fruit matrix. In the absence of fungal growth, this decline is likely driven by intrinsic matrix related processes, including chemical instability and fruit-mediated biochemical reactions occurring within the cherry tomato tissues. The core structure of TeA is characterized by a five membered heterocyclic tetramic acid ring comprising an amide nitrogen and two carbonyl groups [34,35]. This ring forms a conjugated b-dicarbonyl system that allows electron delocalization and exists in equilibrium between keto and enol tautomeric forms [36]. The resulting polarized enol species are readily protonated under mildly acidic conditions, such as the pH acidic environment of tomato cherry tissues [37], thereby increasing susceptibility to acid catalyzed hydrolytic ring opening and progressive loss of the parent compound. In addition, in its enolized form, the tetramic ring provides adjacent oxygen donor atoms capable of coordinating transition metal ions, such as iron (Fer), which are known to be present in tomato tissues [38], forming stable bidentate complexes [39]. Metal coordination may promote localized redox cycling [40], leading to oxidative destabilization of the tetramic ring. In addition, polyphenol oxidase activity (PPO) in tomato tissues generates reactive oxygen species through the oxidation of endogenous phenolics, supplying hydrogen peroxide and other oxidants [41] that can further fuel metal ions to mediated redox cycling. This combined oxidative stress perturbs electron delocalization, weakens the C-N bond, and further favors hydrolytic ring opening, resulting in irreversible loss of intact TeA. Different from TeA, AOH and AME are phenolic dibenzopyrone compounds characterized by a planar aromatic scaffold composed of fused benzene rings linked by a lactone moiety [35]. The presence of phenolic hydroxyl groups introduces highly reactive sites that are prone to oxidation. Accordingly, in tomato tissues, polyphenol oxidase activity may directly target these phenolic moieties, promoting oxidative transformation and contributing to the observed decline of the parent compounds [42]. Beyond oxidation, these hydroxyl functionalities can also associate for fruit’s phase II conjugation pathways. In tomato tissues, endogenous detoxification mechanisms may catalyze glycosylation or sulfation of phenolic xenobiotics, leading to the formation of more polar conjugates [43,44]. Such conjugation reactions reduce chemical reactivity and promote sequestration within plant compartments, for example, as glucosylated or sulfated derivatives of AOH and AME, thereby contributing to the observed decrease in detectable parent AOH and AME [43]. These conjugated compounds have also been reported in the study by Qin et al. [14].

Additionally, it is important to recognize that the toxin concentrations detected during fungal infection likely reflect a net result of production and degradation. While *Alternaria* actively synthesizes toxins during colonization, these compounds may also undergo chemical or enzymatic degradation, either by fungal enzymes or through interactions with the tomato matrix. Thus, the measured toxin levels represent a dynamic balance between ongoing biosynthesis, degradation, and limited diffusion. Understanding this balance is essential for accurately assessing contamination risks in mold-affected produce.

In visibly colonized zones, DNA concentrations ranged from approximately 4000 to over 1,000,000 copies per gram of cherry tomato tissue. These values are consistent with the sensitivity reported in previous studies [45,46], where real-time RT-PCR assays targeting rRNA were used. Although rRNA-based methods generally offer higher absolute sensitivity due to the greater number of rRNA transcripts per cell, the DNA based qPCR approach employed in this study provides a reliable measure of total fungal biomass, enabling detection of both viable and non-viable cells, making it a robust tool for evaluating total contamination. Notably, fungal DNA was detected in distant layers lacking visible fungal growth, such as section S4 and S5 of the sample at the rotten morphological level (Figure 3a), with levels around 5000 copies Alt DNA/g of contaminated cherry tomato. This limited but measurable fungal presence suggests active tissue invasion or early subclinical colonization in areas where the matrix was visibly degraded. These findings underscore that visual inspection alone is insufficient to assess contamination risk. Mycotoxins, and, in some cases, fungal biomass, can be present in seemingly intact tissues, posing significant food safety concerns, particularly in domestic settings where consumers may only discard visibly moldy sections.

**Figure 6:**
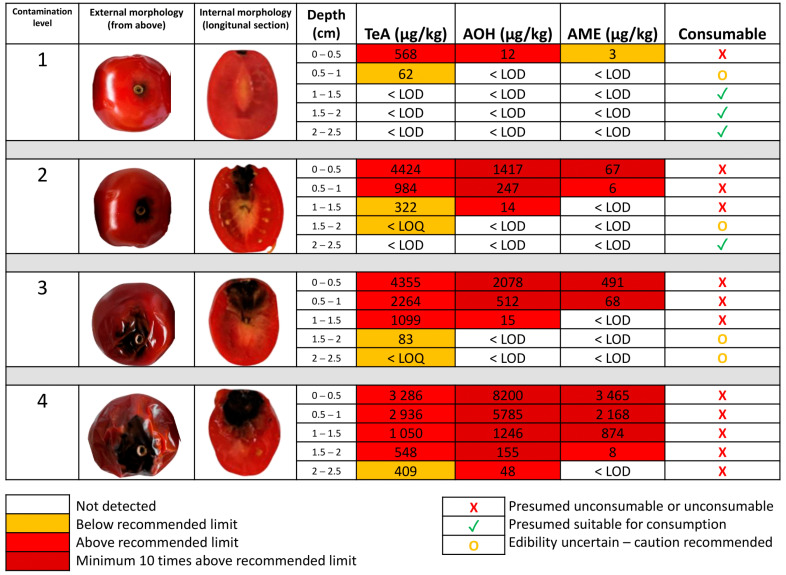
Recommendation for the consumption of cherry tomatoes contaminated by *Alternaria alternata* based on morphological classification and mycotoxin migration (Tenuazonic acid—TeA; Alternariol—AOH; Alternariol monomethyl ether—AME).

Based on the experimental findings, including visual observations, DNA quantification of fungal growth, and analysis of *Alternaria* toxin migration, based on the EU recommendation for *Alternaria* toxins in tomato-based products [20], during both active colonization and passive diffusion, a set of consumer recommendations has been developed to ensure food safety and reduce waste when encountering moldy cherry tomatoes (Figure 6). It is also recommended that consumers cut open the fruit to assess the internal progression of mold, in conjunction with the external morphological signs, to better evaluate the contamination level.

The recommendations were developed using the worst-case scenario in all data collected and applying the indicative levels set by EU Recommendation (2022/553) as provisional thresholds for edibility [20]. However, these indicative levels should be interpreted with caution, as they do not represent safety limits. Ideally, thresholds based on the TTC (Threshold of Toxicological Concern), as employed in EFSA risk assessments, would provide a more robust basis [20]. A maximum allowable toxin concentration in tomatoes (Cmax) can be estimated using the formula:

$$∁max=\frac{TTC\times body\;weight}{Portion}$$

Assuming a 60 kg adult and a highly overestimated daily tomato intake of 0.2 kg (considering tomatoes as the unique source of *Alternaria* toxin exposure), the resulting Cmax values are 450 µg/kg for TeA and 0.75 µg/kg for both AOH and AME. Although the analytical method used meets the sensitivity requirements of EU Recommendation 2022/553 [15,20] (LOQs < 4 µg/kg for AOH and AME, and <20 µg/kg for TeA), it does not achieve the calculated threshold of 0.75 µg/kg for AOH and AME. Consequently, the indicative levels from EU Recommendation 2022/553 [20] were applied, despite their questionable relevance. All concentration data are provided in Figure 6 to allow future refinement of these recommendations as new toxicological data become available.

For the first level (early contamination stage), calculations were based on *A. alternata* toxin production data obtained at day 14 under 8 °C storage conditions. Safe consumption was determined to begin from section S3, located at least 1 cm from the visible colony, as TeA was detected in section S2 at concentrations ranging from 38.03 to 148.63 µg/kg matrix, with no further detection in more distal sections. No evidence of toxin diffusion beyond this point at comparable concentrations was observed. For level 2 (spreading stage), calculations were based on *A. alternata* toxin production data obtained at day 7 under 25 °C storage conditions. Safe consumption was restricted to section S5, located approximately 1 cm from the visible colony and 1–1.5 cm from the inoculation point. This restriction was applied because TeA was detected up to section S4 at low concentrations, with no evidence of further diffusion beyond this section. Level 3 (invasive stage) was considered unsuitable for safe consumption, as *A. alternata* toxins were detected in almost all sections, with TeA present throughout the entire matrix, corresponding to the data model at day 10 under 25 °C incubation. Level 4 (rotten stage) was considered unsafe for consumption. At this stage, corresponding to *A. alternata* toxin production data obtained at day 14 under 25 °C storage conditions, the entire matrix exhibited high concentrations of all monitored *Alternaria* toxins.

Recent reviews have highlighted a major gap in feed-to-fork risk assessment frameworks, namely the limited consideration of human exposure dynamics at the consumer stage, particularly under changing environmental conditions [47]. While predictive models increasingly integrate climate-driven variables affecting fungal growth and mycotoxin production at the crop and feed levels, post-purchase storage and household handling are generally treated as static or negligible components of exposure [47]. The present study directly addresses this gap by experimentally characterizing the spatial and temporal evolution of *Alternaria alternata* toxin distribution within a fresh produce matrix during domestic storage. By linking storage temperature, contamination stage, and distance from visible fungal growth to measurable toxin concentrations, this work provides empirical evidence for how downstream feed-to-fork processes can substantially modify consumer exposure independently of upstream contamination levels. Importantly, the recommendations proposed in this study are not intended to serve as alternatives to HACCP-based control measures in industrial food production or processing. HACCP systems rely on preventive controls, critical control points, and continuous monitoring implemented upstream along the food chain [48], whereas the present framework addresses downstream, consumer-level exposure following product purchase. Its aim to prevent contamination rather than to manage its consequences at the consumer stage. For the proposed recommendations to be translated into industrial risk management tools, substantial additional data and validation steps would be required. These include large-scale occurrence studies encompassing multiple cultivars, fungal strains, production systems, and storage conditions, as well as integration across the entire feed-to-fork chain [49,50]. Moreover, industrial application would necessitate the definition of HACCP-compatible critical limits anchored in toxicological reference values and supported by harmonized analytical methods [51]. Until such data are available, the present recommendations should be considered as consumer-oriented guidance and as experimental input for future exposure modelling rather than as operational criteria for food business operators.

## 4. Conclusions

The study raises further hypotheses about the mechanisms underlying mycotoxin spread. It is plausible that active fungal degradation of host tissues facilitates toxin movement by breaking down structural barriers. Alternatively, the low levels of *Alternaria alternata* DNA detected beyond the visibly colonized zone may reflect early colonization events sufficient to produce toxins locally. Future studies employing histology, micro-imaging, and localized metabolite profiling are needed to clarify whether matrix breakdown or subclinical colonization primarily drives this phenomenon. Importantly, those experimental data, when combined with the consumer-oriented recommendations developed in this study, would provide a foundation for more accurate visual risk assessment of *A. alternata*-contaminated cherry tomatoes at the household level. In the longer term, such evidence-based guidance could contribute to the development of pragmatic safety frameworks that both protect consumers and support food waste reduction strategies.

## 5. Materials and Methods

### 5.1. Isolation, Culture Conditions and Molecular Identification of an Alternaria Strain

An *Alternaria* strain was isolated from a mold-infected cherry tomato sample provided in the MYNION project’s MT292 sampling kit (https://anr.fr/Projet-ANR-22-CE21-0003, accessed on 13 January 2025) from UMR Qualisud, University of Montpellier, Montpellier, France. The isolate was initially cultured on malt extract agar (MEA) and incubated at room temperature for seven days to promote sporulation and mycelial growth. The strain was then resuspended in physiological water and re-incubated for an additional seven days to enhance conidial production. After incubation, a fragment of fungal biomass corresponding to a colony surface area of 1 cm^2^ was collected for genomic DNA extraction. The isolate was maintained on MEA plates until its molecular identification was confirmed.

Genomic DNA was extracted using a modified CTAB protocol. The fungal material, including spores and mycelium, was transferred into a 2 mL microcentrifuge tube containing 500 µL of CTAB lysis buffer containing 5 mL CTAB, 100 µL β-mercaptoethanol, and 0.2 g polyvinylpyrrolidone and mixed with glass beads (425–600 µm). The CTAB extraction buffer consisted of 10% (*v*/*v*) Tris–EDTA buffer (1 M Tris-HCl, 0.1 M EDTA), 28% (*v*/*v*) NaCl (5 M), and 2% (*w*/*v*) cetyltrimethylammonium bromide (CTAB/hexadecyltrimethylammonium bromide). The volume was adjusted to 100% with distilled water. Immediately before use, the buffer was supplemented with β-mercaptoethanol at 2% (*v*/*v*) and polyvinylpolypyrrolidone (PVPP) at 40 mg/mL. Samples were vortexed for 2 min, then incubated at 65 °C for 15 min, vortexed again, and incubated for an additional 15 min. Following lysis, 500 µL of chloroform was added, and the tubes were centrifuged at 13,000× *g* for 5 min. The aqueous phase (400 µL) was recovered and mixed with 200 µL of isopropanol and 40 µL of 3 M sodium acetate (pH 5.2), followed by incubation at room temperature for 1 h and centrifugation at 13,000× *g* for 30 min. The DNA pellet was washed with cold ethanol, recentrifuged, dried, and resuspended in 50 µL of nuclease free water.

PCR amplification was performed to identify the strain using two gene targets: gpd and Alt a1. The gpd gene provides sufficient sequence variability to resolve species within the *Alternaria* section. The Alt a1 gene, which encodes a genus-specific allergen, offers high specificity for *Alternaria alternata* and serves as a confirmatory marker. Primer pairs gpd1/gpd2 and Alt-for/Alt-rev were used, as described by [52,53]. Each 25 µL PCR reaction included 1 µL of DNA, 12.5 µL of AmpliTaq Gold master mix, 1 µL of each primer (10 µM), and 9.5 µL of molecular-grade water. Thermal cycling conditions consisted of an initial denaturation at 94 °C for 10 min, followed by 30 cycles of 95 °C for 30 s, 60 °C for 45 s, and 72 °C for 45 s, with a final elongation at 72 °C for 7 min. Amplicons were separated by electrophoresis on 1.5% agarose gels stained with ethidium bromide and visualized under UV light. The presence of DNA bands (about 500–600 bp for gpd, and 400–500 bp for Alt a1) confirmed successful amplification. PCR products were then submitted for Sanger sequencing (Eurofins Genomics, Ebersberg, Germany).

After molecular confirmation as *Alternaria alternata*, the strain was preserved at −80 °C in cryobead vials to maintain genetic integrity and reduce phenotypic variation caused by repeated subculturing [54,55].

### 5.2. Fungal DNA Quantification

Cherry tomatoes inoculated with *Alternaria alternata* were ground in liquid nitrogen, and 200 mg of homogenized tissue were transferred into MN Type E bead tubes. DNA extraction was performed on the Beckman BIOMEK^®^ FX robotic workstation at GPTR (Great regional technical platform, CIRAD, Montpellier, France) using the NucleoMag^®^ DNA Microbiome Kit (Macherey-Nagel GmbH & Co. KG, Valencienner Str. 11, Düren, Germany). Fungal DNA quantification was carried out by qPCR on the MGX-Montpellier GenomiX platform of University of Montpellier, Triolet Campus, Montpellier, France.

Fungal DNA was quantified by TaqMan qPCR using primers Alt F (5′-TCTTTTGCGTACTTCTTGTTTCCTT-3′) and Alt R (5′-TTACTGACGCTGATTGCAATTACA-3′), along with probe Alt P (5′-TGGGTTCGCCCACCACTAGGACA-3′) [53], targeting a 90 bp fragment (OP758812.1).

The reaction mix, containing each primer at 0.4 µM, the probe at 0.2 µM, SensiFast Probe No-Rox Kit 2X (Bioline, Unit 16, The Edge Business Centre, Humber Road, London, UK) was introduced in Echo Qualified 384-well Polypropylene Source Microplate, as well as DNA extracts. A volume of 0.5 µL of diluted DNA extract and 1 µL of reaction mix was transferred from the source plate to the qPCR plate using the EchoR525 Liquid Handler V2.6 and the Echo Plate Reformat software V1.7.2. PCR was performed on a LightCycler^®^ 480 instrument (Roche Diagnostics GmbH, Mannheim, Germany) using the LightCycler^®^ 480 software version 1.5. The thermal cycling program consisted of an initial denaturation at 95 °C for 3 min, followed by 45 cycles of denaturation at 95 °C for 10 s and annealing/extension at 60 °C for 40 s. DNA from *Alternaria* spores quantified using a Qubit 2.0 fluorometer (Invitrogen, Thermo Fisher Scientific, 168 Third Avenue, Waltham, MA, USA) was used to generate a standard curve, and the number of copies was determined by droplet digital PCR (ddPCR). The ddPCR reaction mixture consisted of 2 µL of DNA extract, molecular biology–grade water to reach 21/40 of the final volume, 1 µL each of primers Alt F and Alt R, 3/40 of the final volume of EvaGreen 20X (Biotium, Biotium, Inc., Hayward, CA, USA), 1 µL of Alexa Fluor 647 at 0.02 g/L (ThermoFisher, Thermo Fisher Scientific, 168 Third Avenue, Waltham, MA, USA), and 5 µL of PerfeCTa 5X (VWR International, Radnor, Pennsylvania, PA, USA). Droplets were generated on Sapphire chips V4.0, thermocycled under the same conditions as the qPCR, and analyzed with the Prism3 reader (Stilla Technologies, Villejuif, France). Quantification was calibrated using DNA standards measured with a Qubit fluorometer, with the qPCR detection limit set at Cp = 32, corresponding to approximately 10^3^ copies per gram of cherry tomato tissue.

The quantification results are expressed as the number of *A. alternata* DNA fragment copies per gram of cherry tomato matrix (Alt DNA copies/g).

### 5.3. Preparation of Conidial Suspension

Spore suspensions of *Alternaria alternata* were obtained from 10-day old cultures grown on modified malt extract agar (MEA; 20 g/L malt extract, 1 g/L peptone, 20 g/L glucose, 15 g/L agar), optimized for sporulation. To harvest spores, 10 mL of sterile physiological water with 1% Tween 80 was added to each plate, and spores were gently dislodged using a sterile L-shaped spreader (Biologix Group Limited, Jinan, China). The suspension was then filtered through sterile cotton to eliminate mycelial debris. Spore concentration was determined using a Thoma hemocytometer (Hirschmann Laborgeräte GmbH & Co. KG, Bad Blankenburg, Germany) and adjusted to 1 × 10^6^ spores/mL for use in all inoculation experiments.

### 5.4. In Vitro Toxin Production Assay

Toxin production by *Alternaria alternata* was assessed in vitro using a spore suspension at 1 × 10^6^ spores/mL. A 10 µL droplet of the suspension was inoculated at the center of Petri dishes containing one of three culture media: MEA, PDA (potato dextrose agar) or TCA (tomato coulis agar) prepared from 60% (*w*/*w*) diluted organic tomato coulis free from citric acid or NaCl, and 15 g/L agar.

Plates were incubated at 25 °C, and analyses were performed at 3, 7, 10 and 14 days, in triplicate. At each time point, the colonies were photographed, and the colony diameter and surface area were measured using ImageJ (V64-bit Java 8). Half-colonies, along with corresponding half-agar portions, were collected into 50 mL Falcon tubes, weighed, and stored at −20 °C for downstream analyses.

### 5.5. In Vivo Inoculation Model

A spore suspension of *Alternaria alternata* at a concentration of 1 × 10^6^ spores/mL was used to inoculate cherry tomatoes. Prior to inoculation, tomatoes were disinfected by immersion in three successive baths: 70% ethanol for 30 s, 1% sodium hypochlorite for 5 min, and distilled water for 2 min. They were then air-dried.

To determine the optimal site for tracking *A. alternata* migration, preliminary inoculations were performed at three locations on the cherry tomato surface: the pedicel scar, equator, and stylar scar. Since fungal progression can vary depending on the inoculation site, these tests helped identify the most suitable entry point.

Based on this comparison, an inoculation site was selected, where a 2 mm diameter wound was made using a sterile pipette tip to facilitate fungal entry. A volume of 10 µL of the spore suspension was applied directly into each wound, in triplicate (3 repeated experiments for 6 fruits). Two storage conditions were used to simulate different postharvest environments: incubation at 8 °C represented a cellar or eco-mode refrigerator, while incubation at 25 °C reflected typical ambient conditions.

**Figure 7:**
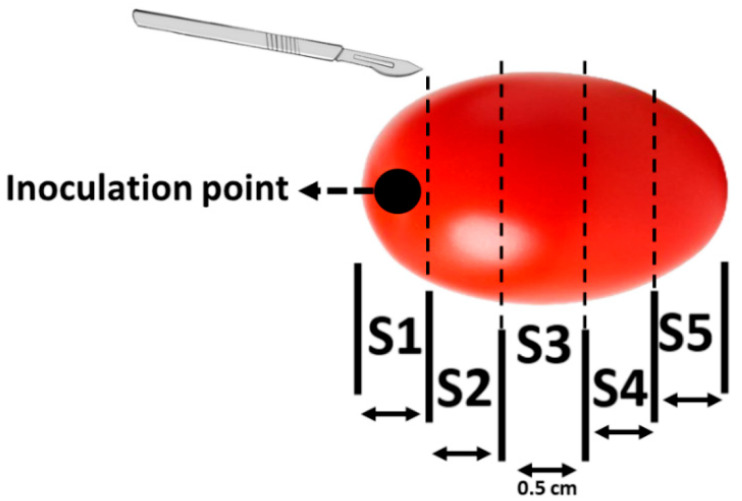
Schematic of tomato cross-sectioning for layer-specific analysis (S*n*: Section order).

Tomatoes were grouped for each incubation period: 3, 7, 10 and 14 days, based on the morphology degree of contamination, evaluated using ImageJ. After incubation, the fruits were frozen at −20 °C for 24 h to facilitate precise sectioning. Each fruit was then sliced into 0.5 cm-thick cross-sections starting from the stylar scar area (Figure 7). All slices from each tomato were pooled into 50 mL Falcon tubes and homogenized using an Ultra-Turrax^®^ homogenizer (T25, IKA-Werke GmbH & Co. KG, Staufen, Germany). Homogenates were stored at −20 °C or immediately processed for downstream analyses.

### 5.6. Artificial Toxin Contamination Model

In parallel with the in vivo inoculation model, a 10 µL of solution containing known concentrations of the three target mycotoxins prepared with Standard powders (1 mg) of AOH, AME, TeA from Libios (Vindry sur Turdine, France), was directly injected into the cherry tomatoes to assess the passive diffusion of mycotoxins in the absence of fungal growth. The injection model was in triplicate (3 repeated experiments for 6 fruits), followed the same inoculation point, incubation conditions, time points, and sample processing protocols as described in the in vivo inoculation model.

### 5.7. Mycotoxin Extraction and Quantification

For mycotoxin extraction and quantification method, 3 g of the homogenized sample were processed using standardized extraction protocols for subsequent chromatographic analysis [15]. Mycotoxin extraction was performed as follows: 7 mL of acetonitrile and 1.5 mL of a water/acetic acid solution (89/11, *v*/*v*) were added to Falcon tubes containing the half-colonies, followed by agitation for 10 min. Subsequently, 2 g of NaCl was introduced to induce phase separation between the aqueous phase and acetonitrile, facilitating the migration of mycotoxins into the non-polar acetonitrile phase, which enhances extraction efficiency. The Falcon tubes were then agitated for another 10 min and centrifuged at 9000× *g* for 10 min at 4 °C.

After centrifugation, 5 mL of the acetonitrile supernatant (containing the extracted mycotoxins) was collected and mixed with 3 g of anhydrous Na_2_SO_4_ to remove any residual water. A second centrifugation step was performed (10 min at 9000× *g*, 4 °C), after which 300 μL of the supernatant was transferred into vials. In each vial, the following were added: 100 μL of acetonitrile, 700 μL of mobile phase A (water/acetic acid: 99.5/0.5) and 100 μL of a C13-labeled internal standard solution at 1 μg/mL. Finally, the sample content was filtered through 0.45 μm PTFE filters, after which the filtered extracts were injected and analyzed by LC-MS/MS.

Quantification of TeA, AOH, and AME, was performed using a Shimadzu LCMS-8040 triple quadrupole mass spectrometer (Shimadzu, Kyoto, Japan) equipped with an electrospray ionization (ESI) source operating in both positive and negative modes. Chromatographic separation was carried out on a reversed-phase Kinetex C18 column (2.6 µm, 100 Å, 50 × 2.1 mm, Phenomenex, Sec.2, Taipei City, Taiwan) equipped with a C18 pre-column (2.1 mm) maintained at 50 °C. The injection volume was 25 µL. The mobile phase consisted of water with 0.5% acetic acid (phase A) and isopropanol with 0.5% acetic acid (phase B), delivered at a flow rate of 0.4 mL/min according to the following gradient: the first 4 min and 15 s, the gradient decreased from 95% A to 10% A. This composition (10% A) was maintained until 5 min and 47 s. Subsequently, the gradient returned to 95% A until 10 min.

Mass spectrometric detection was conducted under the following conditions: nebulizing gas flow 2.5 L/min, drying gas flow 15 L/min, desolvation line temperature 125 °C, heater block temperature 350 °C, and collision-induced dissociation (CID) gas pressure 230 kPa using argon. Quantification and confirmation were based on multiple reaction monitoring (MRM) transitions. The quantifier (Q) and qualifier (q) MRM used were as follows: for AOH (−) 257.0 → 215.0 (Q) and (−) 257.0 → 170.95 (q); for AME (−) 271.2 → 256.0 (Q) and 271.0 → 228.0 (q); for TeA (−) 196.0 → 139.1 (Q) and (−) 196.0 → 112.05 (q). An isotopically labeled internal standard of AOH-C13 (−) 271.05 → 225.0 was used for internal calibration of AOH and AME. Quantification was based on the Q transition, while the q transition was used for confirmation of analyte identity. The limits of quantification (LOQ) for the targeted mycotoxins were determined as follows: 3.5 µg/kg for AOH, 2.5 µg/kg AME, and 18.4 µg/kg for TeA.

### 5.8. Statistical Analysis

Statistical analyses of toxin concentrations in both in vitro and in vivo models (with and without mold presence), as well as fungal DNA copy numbers, were conducted to assess whether significant changes occurred over the course of colony size evolution, across tissue layers, and under all storage conditions. Given the non-normal distribution and heteroscedasticity of the data, the non-parametric Kruskal–Wallis test was selected as the most appropriate method. This test evaluates whether the rank distributions of measured values differ significantly between sampling levels. When significant global differences were detected, Dunn’s post hoc test with Bonferroni correction was subsequently applied to determine which specific groups differed from one another. The following thresholds were used for interpreting *p*-values: *p* ≤ 0.001 indicates very highly significant results (strong evidence); 0.001 < *p* ≤ 0.01, highly significant; 0.01 < *p* ≤ 0.05, significant at the 95% confidence level; 0.05 < *p* ≤ 0.1, marginally significant (trend); and *p* > 0.1, not significant.

Z-score normalization of toxin to DNA ratios was applied to evaluate toxin production activity corresponding of fungal biomass. For each sampling day and section, the ratio of toxin concentration to fungal DNA was transformed into a z-score, allowing comparison of relative toxin production across tissues with different levels of colonization. Positive z-scores indicate toxin production levels higher than the mean relative to fungal biomass, reflecting enhanced toxigenic activity, whereas negative z-scores indicate lower-than-average toxin production relative to fungal DNA. Z-scores close to 0 represent toxin production proportional to fungal biomass.

All analyses were conducted using R language (V4.5.1).

## Figures and Tables

**Table 1:** *Alternaria alternata* toxin production (TeA, AOH, AME) on three different agar medium (MEA, PDA, and TCA) after 3, 7, and 10 day post-inoculation. Data are min-max values from 3 biological replicates.

		Medium		
Toxin (µg/cm^2^)	Day	MEA	PDA	TCA
	3	2.98–6.55 (b) ^1^	3.63–8.03 (b) ^1^	6.70–12.17 (b) ^1^
TEA	7	11.74–24.11 (bc) ^1^	6.95–10.11 (b) ^1^	19.05–35.62 (c) ^1^
	10	31.20–49.24 (cd) ^1^	30.06–68.86 (cd) ^1^	18.88–26.29 (c) ^1^
	3	<LOQ–0.01 (a) ^1^	0.09–0.27 (ab) ^1^	0.01–0.03 (a) ^1^
AOH	7	0.09–0.38 (ab) ^1^	0.12–0.25 (ab) ^1^	0.08–0.23 (ab) ^1^
	10	19.79–26.84 (c) ^1^	0.71–0.89 (b) ^1^	0.52–1.11 (b) ^1^
	3	<LOQ	0.02–0.08 (a) ^1^	<LOQ
AME	7	<LOQ–0.01 (a) ^1^	0.25–0.46 (ab) ^1^	0.01–0.06 (a) ^1^
	10	9.94–15.56 (bc) ^1^	0.16–0.22 (ab) ^1^	0.08–0.20 (ab) ^1^

^1^ Letters indicate statistically significant differences between toxin concentration data in different timeline, based on Kruskal–Wallis followed by Dunn’s post hoc test (*p* < 0.05).

**Table 2:** Visual assessment of *Alternaria alternata* infection of the in vivo kinetic model in cherry tomatoes at 25 °C and 8 °C.

DPI	8 °C	25°C	Depth (cm)	Section
3	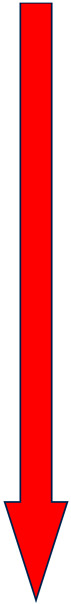	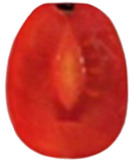	0–0.5	S1
			0.5–1	S2
			1–1.5	S3
			1.5–2	S4
			2–2.5	S5
7		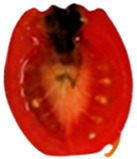	0–0.5	S1
			0.5–1	S2
			1–1.5	S3
			1.5–2	S4
			2–2.5	S5
10		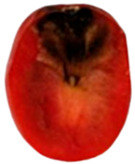	0–0.5	S1
			0.5–1	S2
			1–1.5	S3
			1.5–2	S4
			2–2.5	S5
14	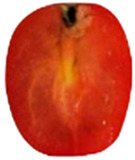	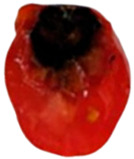	0–0.5	S1
			0.5–1	S2
			1–1.5	S3
			1.5–2	S4
			2–2.5	S5

**Table 3:** Visual classification of *Alternaria alternata* infection in cherry tomatoes.

Contamination Level	External Morphology ^1^	Internal Morphology ^2^
1 Early contamination	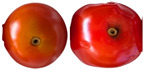	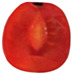
2 Spreading	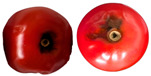	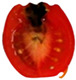
3 Invasive	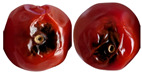	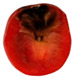
4 Rotten	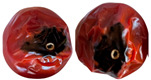	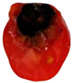

^1^ From above; ^2^ Longitudinal section.

## Data Availability

The original contributions presented in this study are included in the article. Further inquiries can be directed to the corresponding authors.

## Abbreviations

The following abbreviations are used in this manuscript:

A	Mobile Phase A
AOH	Alternariol
AME	Alternariol Monomethyl Ether
B	Mobile Phase B
bp	Base Pair(s)
CID	Collision-induced Dissociation
CIRAD	French Agricultural Research Centre for International Development
cm	Centimeter
cm^2^	Square Centimeter
cm^3^	Cubic Centimeter
dpi	Post-inoculation day
CTAB	Cetrimonium Bromide
DNA	Deoxyribonucleic Acid
EU	European Union
FAO	Food and Agriculture Organization of the United Nations
g	Gram
gpd	Glyceraldehyde-3-phosphate Dehydrogenase
HACCP	Hazard Analysis Critical Control Point
Kg	Kilogram
kPa	Kilopascal
LC-MS/MS	Liquid Chromatography With Tandem Mass Spectrometry
LOQ	Limit Of Quantification
MEA	Malt Extract Agar
Min	Minute
MN	Macherey-Nagel
MRM	Multiple Reaction Monitoring
NaCl	Sodium Chloride
Na_2_SO_4_	Sodium Sulfate
PDA	Potato Dextrose Agar
PCR	Polymerase Chain Reaction
pKW	*p*-value Kruskal–Wallis
PTFE	Polytetrafluoroethylene
q	Qualifier Transition
Q	Quantifier Transition
qPCR	Quantitative Polymerase Chain Reaction
rRNA	Ribosomal Ribonucleic Acid
R	Pearson Correlation Coefficient
RT-PCR	Real-time Polymerase Chain Reaction
S(n)	Section (number)
TCA	Tomato Coulis Agar
TeA	Tenuazonic Acid
µg	Microgram
µL	Microliter
UV	Ultraviolet
mm	Millimeter
